# Systematic Study of Immune Cell Diversity in ischemic postconditioning Using High-Dimensional Single-Cell Analysis with Mass Cytometry

**DOI:** 10.14336/AD.2020.1115

**Published:** 2021-06-01

**Authors:** Yang Yao, Yaning Li, Weihua Ni, Zhijun Li, Liangshu Feng, Yan Wang, Jihong Meng, Heng Zhao

**Affiliations:** ^1^Department of Neurosurgery, Stanford University School of Medicine, Stanford, CA 94305, USA; ^2^Division of Plastic and Reconstructive Surgery, Department of Surgery, Stanford University School of Medicine, Stanford, CA 94305, USA

**Keywords:** ischemic postconditioning, mass cytometry, immune cell diversity, ischemic brain, peripheral blood

## Abstract

Ischemic postconditioning (IPostC) is a concept of ischemic stroke treatment, in which several cycles of brief reocclusion after reperfusion are repeated. It is essential to have an accurate understanding of the immune response in IPostC. By using high parametric single-cell mass cytometry, immune cell subsets and characterize their unique functions from ischemic brain and peripheral blood were identified after IPostC. This study enabled us to better understand the immune cell phenotypical and functional characteristics in ischemic brain and peripheral blood at the single-cell and protein levels. Since some cell surface markers can serve as functional markers, reflecting the degree of inflammation, the cell surface marker intensity among different groups was analyzed. The results showed that downregulation of 4E-BP1 and p38 of Microglia and MoDM in the ischemic brain was involved in IPostC-induced protection. In the peripheral blood, downregulation of P38 of CD4 T cell and Treg has also participated in IPostC-induced protection.

Inflammation and immunity are crucial elements to understanding the pathobiology of stroke, which is a destructive illness. Stroke is one of the leading causes of death worldwide. The immune system involved in the ischemic brain damage, in turn, the damaged brain has an immunosuppressive effect that promotes lethal infections that threaten people’s survival after stroke [1]. Ischemic postconditioning (IPostC) refers to a series of transient occlusions of cerebral blood vessels after reperfusion, which is an emerging concept for stroke treatment [2-4]. Our previous studies have shown that IPostC promoted the decrease of brain infarction size and alleviated neurological deficits in mice [5] and rats [4, 6-10], probably by blocking both innate and adaptive immune cells infiltrated into the ischemic brain [5]. In addition, peripheral lymphopenia was actively attenuated by IPostC and thus improved systemic immunodepression. Thus, IPostC exerts a prominent protective effect on the ischemic brain and offers new stroke therapies [5]. Also, our previous studies have demonstrated that some signaling events involved in IPostC, which has shown to play a vital role in IPostC-induced protection. There are targeting classic immune signaling events such as serine-threonine kinase Akt [11, 12], mechanistic target of rapamycin (mTOR) [13] and mitogen-activated protein kinase (MAPK) [14] pathway, which improves the outcomes after IPostC. However, we are not entirely understood the regulatory functions of immune factors and their corresponding underlying mechanisms. In this study, our focus was to analyze the effects of IPostC on the potential link between immune cells and signaling events in the ischemic brain and peripheral blood.

Despite the importance of immune response in IPostC, our ability so far is limited to characterize immune cell populations and understand their distinct functions fully. In order to identify immune cell populations in brain tissues, most of the studies have relied on flow cytometry, immunohistochemistry, and gene expression assay. While we have been taught a lot by these techniques, they also have some limitations. Mass Cytometry, which is also named Cytometry by time of flight (CyTOF), combines flow cytometry with mass spectrometry of inductively coupled plasma (ICP) [15]. CyTOF uses the same clones of antibodies using in flow cytometry, which conjugated to rare earth metals as reporters. A significant benefit is to avoid the interference of autofluorescence from cells and spectral overlap between dyes used for flow cytometry [16, 17]. CyTOF stands for a step-change to capture phenotypical and functional characteristics of immune cells in such a broad and high-dimensional perspective at the single-cell level [18, 19].

Here, we used CyTOF to broadly identify the immune cell subsets in ischemic brain and peripheral blood after IPostC. Using this approach, we defined the phenotype and characterize in lymphoid and myeloid cell subsets (including T cells, B cells, NK cells, dendritic cells (DCs), monocytes, and macrophages) after IPostC. The evoked intracellular responses were assessed by measuring immune signaling events involved, including the phosphorylation of crucial kinases and transcription factors or total protein levels of regulatory factors.

## MATERIALS AND METHODS

### Mice

Adult (8-10 weeks of age; 20-25 g) male C57BL/6 mice were purchased from The Jackson Laboratory (Bar Harbor, ME, USA). Mice were maintained under specific-pathogen-free (SPF) conditions in veterinary service center of Stanford university. Five mice were kept in a 12:12-h light cycle in each cage. The temperature was set at 65-75°F with 40-60% humidity. All experiments were performed in accordance with the National Institutes of Health’s *Guide for the Care and Use of Laboratory Animals* and in accordance with the ARRIVE (Animal Research Reporting In Vivo Experiments) guidelines. All procedures and protocols were approved by the Institutional Animal Care and Use Committee of Stanford University.

### Middle cerebral artery occlusion (MCAO) and IPostC

Before surgery, anesthesia was induced by 5% isoflurane and maintained at 2% isoflurane during the procedure. The core body temperature was monitored with a rectal probe and maintained throughout the entire procedure at 37±0.5°C with a surface heating system. Focal ischemia was induced as described previously. Briefly, make a ventral midline neck incision under the operating microscope, the left common carotid artery (CCA) and external carotid artery (ECA) were exposed and isolated. The ECA and the CCA distal to its bifurcation were ligated. A loose tie was made around the CCA proximal to its bifurcation and the internal carotid artery (ICA). A small incision was made in the CCA and a 6-0 silicon coated nylon monofilament with a 0.23 mm tip diameter (Doccol, Redlands, CA, USA) passed into it. With the filament inserted, the loose tie around the CCA was secured, then remove the loose tie around the ICA and the filament guided up the ICA until a slight resistance was felt, approximately 9-10mm. Successful occlusion was verified by Laser Doppler. After 60 min induced ischemia, the filament was removed. Sham-operated mice underwent the same procedure, but without monofilament insertion.

To induce IPostC, the suture was withdrawn only about 2 mm to allow brief, repetitive MCA occlusions. IPostC with 3 cycles of 15 sec occlusion/30 sec release started at 2 min after reperfusion. Animals were observed throughout recovery from anesthesia.

### Single-cell suspension preparation

After IPostC, the mice were sacrificed at day 1 and day 3. The ischemic brain and peripheral blood were collected as described below for CyTOF analyses. Before perfusion, blood samples were collected from the caudal vein by using hematocrit tubes, then transfer into an ethylene diamine tetraacetic acid (EDTA)-coated sterile test tube and washed twice with staining buffer. Mice were anesthetized with an overdose of isoflurane and then perfused with ice-cold phosphate-buffered saline (PBS). Ischemic hemisphere was dissected and transferred to RPMI-1640 (Sigma-Aldrich) solution. The homogenate was filtered through a 70 μm Falcon™ cell strainers (BD Falcon, NJ, USA) after being homogenized with the plunger of a syringe. Then gradient centrifugated with 30% and 70% Percoll (Sigma-Aldrich, GE Healthcare Life Sciences, USA). The suspension was centrifuged at 600 x g for 30 min at 4°C with the brake of the centrifuge switched off. The interphase cells were collected by aspiration with plastic pipette and completely washed with RPMI-1640 solution [20]. The cell pellets were resuspended in 1 ml of staining buffer and washed one more time.

### Mass cytometry

First, approximately 3×10^6^ cells from each sample were incubated with Cell-ID Cisplatin (Fluidigm, CA, USA) for 10 min to identify live/dead cells, then washed twice with Maxpar cell staining buffer (Fluidigm, CA, USA). To barcode the samples, they had to be fixed and permeabilized [21]. For fixing, the cells were re-suspended in 1 ml Fix I buffer (Fluidigm, CA, USA) and incubated for 10 min at room temperature, and then centrifuged. The pellets were permeabilized by washing with 1 ml Maxpar barcode perm buffer (Fluidigm, CA, USA). After fixation and permeabilization, the cells were resuspended in 800 µl barcode perm buffer, mixed with the barcodes diluted in 100 µl barcode perm buffer and incubated for 30 min at room temperature. After that, the cells were centrifuged and washed twice with 1 ml of Maxpar cell staining buffer. The cells were resuspended in 50 µl Maxpar cell staining buffer for immunostaining, added with Fc-receptor (BioLegend, CA, USA) blocking solution and incubated for 10 min. Followed by stained each sample with the antibody cocktail (antibody panel shown in Supplementary Table 1) in 100 μl final volume for 30 min at room temperature. The cells were centrifuged and discarded the supernatant. This was followed by incubation with 500 µl intercalating solution (Iridium, Fluidigm, CA, USA) for 1h at room temperature. The cells were washed twice by adding 1 ml of Maxpar cell staining buffer and centrifuged, and once with 1 ml of MilliQ water before acquisition. To normalize the data from different acquisition time, EQ four element beads (Fluidigm, CA, USA) were added and adjusted cell concentration to 2.5-5×10^5^/ml. Then the cells were injected into the CyTOF machine and processed for data acquirement (CyTOF2, Fluidigm, CA, USA).

**Figure 1.**
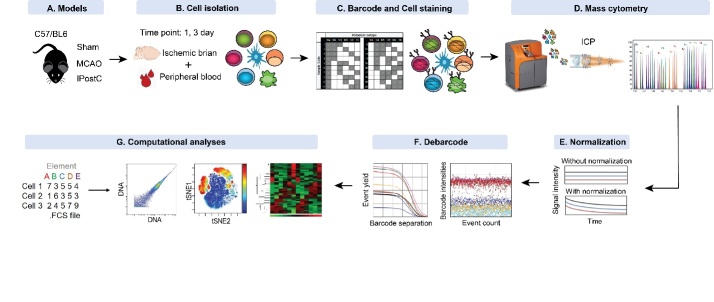
Schematic illustration of experimental design and procedure. (A) C57/BL6 mice were divided into 3 groups, Sham, MCAO, and IPostC. (B) Animals were euthanized for tissue collections at 1, 3 days after stroke, and immune cells were isolated from the ischemic brain hemisphere and peripheral blood. (C) All samples were barcoded by a combination of three palladium (Pd) mass tags. Cells from the same tissue collected at the same time point were pooled, stained using metal-labeled 35 antibodies against cell surface markers, transcriptional factors, and phosphorylated proteins. (D) The samples were analyzed by mass cytometry (CyTOF). The probes are separated on the basis of their mass to charge ratio and plotted with time. (E) Raw mass cytometry data were normalized for signal variation over machine-time using normalization beads containing known concentration of metal isotopes. (F) The combined sample was then loaded into existing matlab-based debarcoding software to automatically assign cells back to their initial identity. In the separation tab, observe the barcode separation plots and choose a minimum barcode separation value just before the event yield dramatically drops. Event plot for barcode population shows clear separation with very few events appearing in the gap which is expected. (G) The CyTOF data was analyzed by manual gating and viSNE. The marker intensity was analyzed using heatmap. ICP: Inductively Coupled Plasma.

### Data processing and analysis

All acquired files in the experiment were normalized and debarcoded using the Helios software. Then we use Cytobank https://www.cytobank.org (Cytobank Inc.) for further data processing and analysis.

To detect clusters of cells with similar expressions of cell surface markers in CyTOF, live CD45^+^ leukocytes were gated, and a selected marker expression on a single cell level was clustered using the unsupervised dimensionality reduction algorithm t-Distributed Stochastic Neighbor Embedding (t-SNE) algorithm^3^ in Cytobank (1,000 iterations, Perplexity 30, Theta 0.5) [22, 23]. The final KL divergence was 1.849. Cell populations were then defined in accordance with standard definitions of cell types based on the expression of markers, which were used are listed in Supplementary Table 2. In each cell subsets, the markers’ intensity levels were calculated on a transformed (arcsinh(X/5)) median value. So, 1 represented the highest expression, and no expression is 0 in the heatmap. According to manual gating, the percentage of major cell subsets was identified.

**Figure 2.**
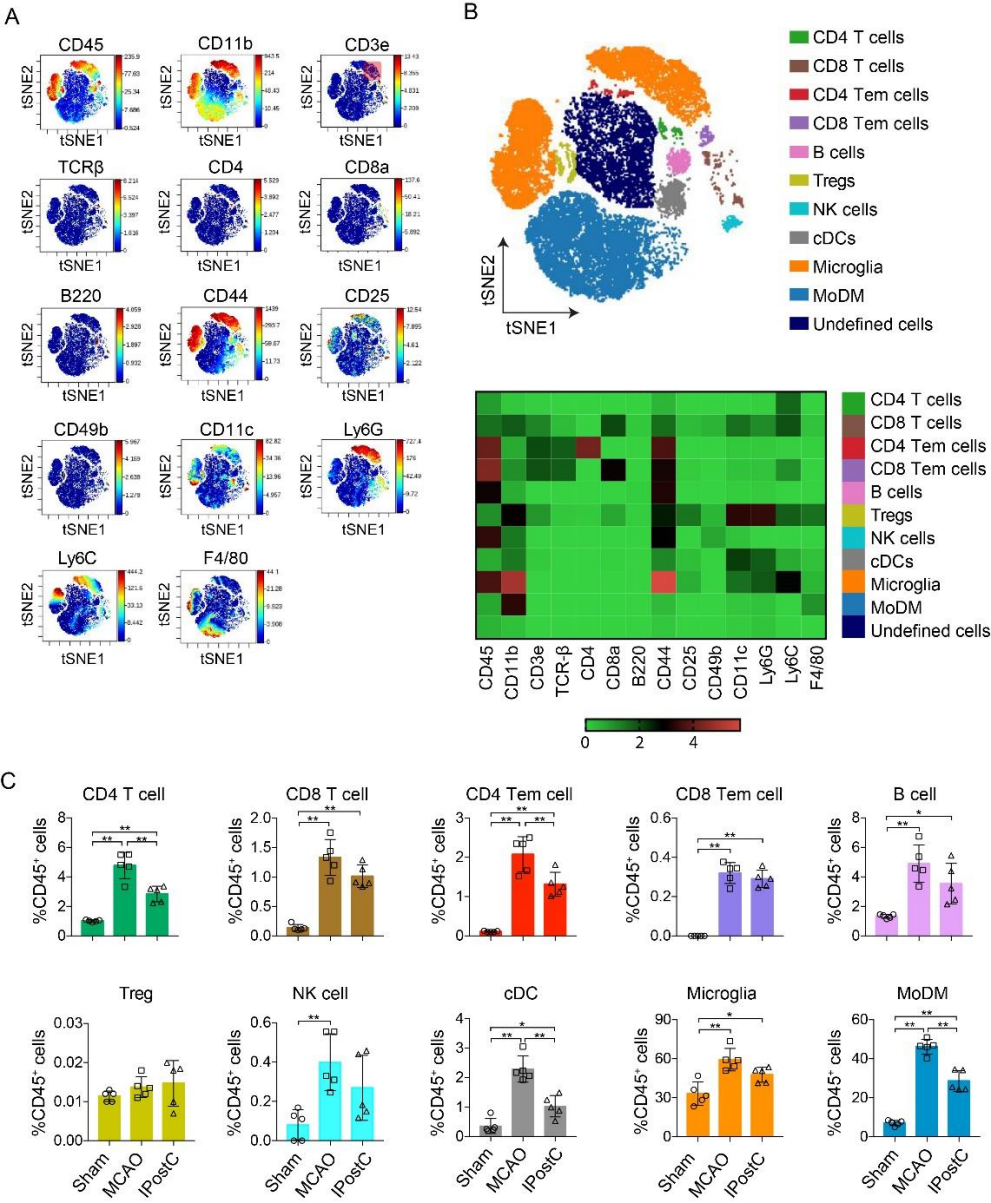
Mass cytometry reveals immune cell subsets in ischemic brain at day 3 after stroke or IPostC. (A) Live CD45^+^ cells concatenated from the ischemic brain of all mice studied (n= 5) were clustered using the viSNE on the expression of 14 cell surface markers. Expression levels of selected markers in the resulting viSNE clustered cell populations are shown. (B) viSNE plots of clustered CD45^+^ cells are displayed for representative mice at day 3 after stroke. The analysis identifies 11 populations including myeloid, lymphocytes, and unknown subsets. (C) Heatmap showing the relative expression level of 14 cell surface markers on the 10 immune cell subsets identified by the viSNE clustering shown in (B). (D) Quantification of immune cell subsets identified in the viSNE clustering outlined in B, between sham, MCAO and IPostC. Data are presented as Mean ± SD, and dots on the bars represent individual samples. MoDMs, monocyte-derived macrophages; CD4Tem/ CD8Tem cell, effector memory CD4 and CD8 T cells. Treg, regulatory T cells; cDC, conventional dendritic cells; NK cells, natural killer cells. n=5/group. *, **, P<0.05, 0.01, respectively, between the two indicated groups.

**Figure 3.**
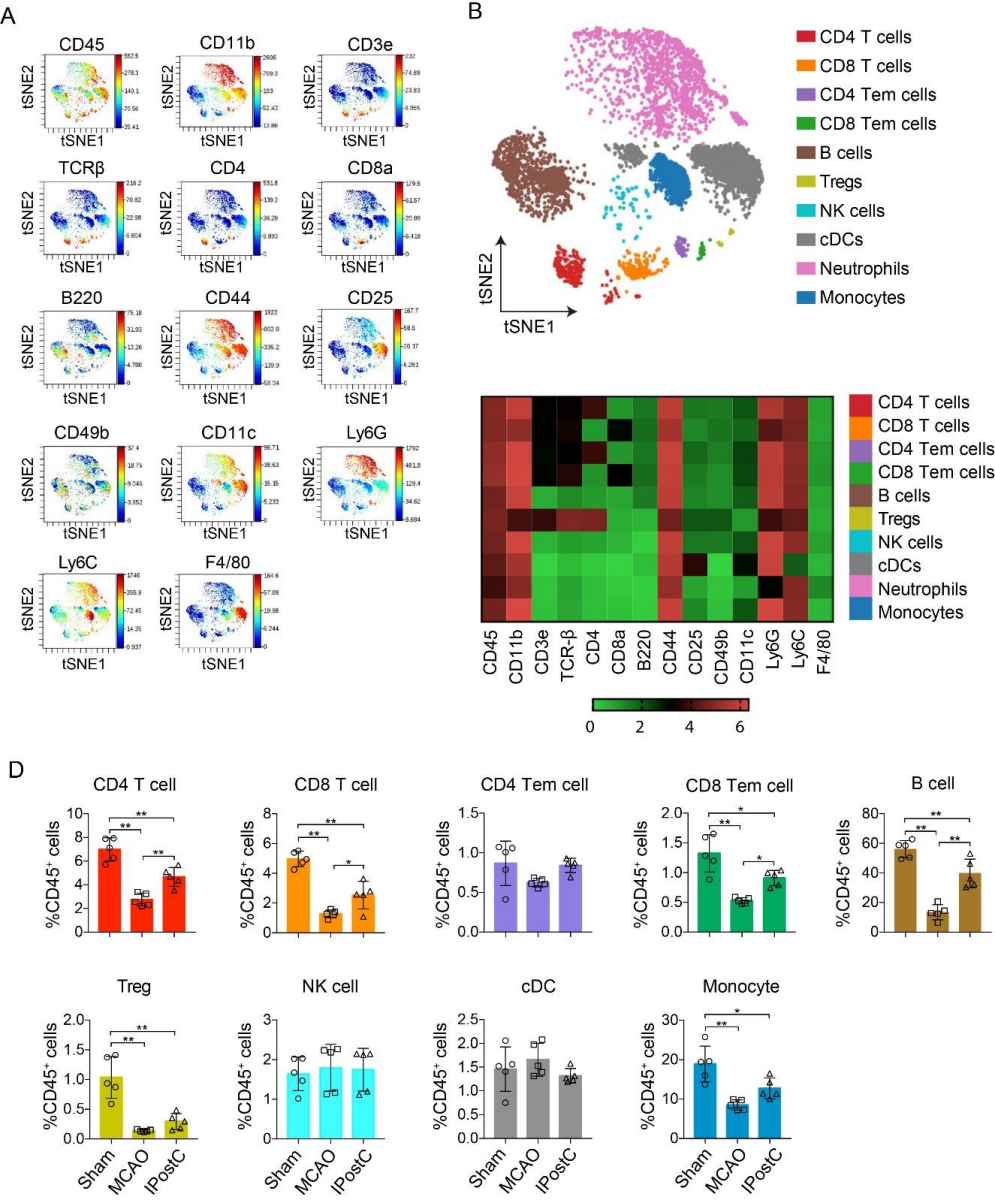
Mass cytometry reveals immune cell subsets in peripheral blood at day 3 after stroke or IPostC. (A) Live CD45^+^ cells concatenated from the peripheral blood of all mice studied (n= 5) were clustered using the viSNE on the expression of 14 cell surface markers. Expression levels of selected markers in the resulting viSNE clustered cell populations are shown. (B) viSNE plots of clustered CD45^+^ cells are displayed for representative mice at day 3 after stroke. The analysis identifies 10 cell populations including myeloid cells and lymphocytes. (C) Heatmap showing the relative expression level of 14 cell surface markers on the 10 immune cell subsets identified by the viSNE clustering shown in (B). (D) Quantification of immune cell subsets identified in the viSNE clustering outlined in B, between sham, MCAO and IPostC. Data are presented as Mean ± SD, Dots represent individual samples, CD4 Tem/ CD8 Tem cell = effector memory T cells/B cells; Treg cells = regulator of T cells; cDC=conventional dendritic cells; NK cells = natural killer cells, n=5/group, *, **, P<0.05, 0.01, respectively. between the two indicated groups.

### Detraction and visualization of differential functional proteins

Differential expression endogenous functional immune features in each cell type were compared between MCAO group and Sham group using ANOVA test. Endogenous functional immune features with two-fold difference and adjust p value less than 0.01 were selected as significant differential expression functional immune features. The expression changes compared to the mean expression of Sham group (n=5) were shown in the heatmap with hierarchical clustering by distance using R package. We next compared the expression change between MCAO group and IPostC group and restricted the comparison in the differential functional immune features. Functional immune features with significant two-fold (p adjust <0.01) difference between MCAO and IPostC group were selected as the IPostC responding functional immune features. The expression changes were shown as a heatmap after normalized to the mean value of Sham group, with hierarchical clustering by distance using gplots package (heatmap.2 function) of R program (version 3.5.1).

### Statistical analysis

Data are expressed as Mean ± SEM. Statistical analyses were performed using GraphPad Prism 7.0 software (Software for Science, San Diego, CA). The sample size calculation was performed at a power of 0.8 and to a significance level of 0.05 by using G*Power software. One-way ANOVA followed by Tukey post hoc test was used for comparisons of three or more groups using GraphPad prism (GraphPad Software, Inc., CA, USA). Two-way ANOVA followed by Bonferroni post-tests was used for multiple comparisons. Differences were considered statistically significance for *p* < 0.05. All the results represent a single experiment.

**Figure 4.**
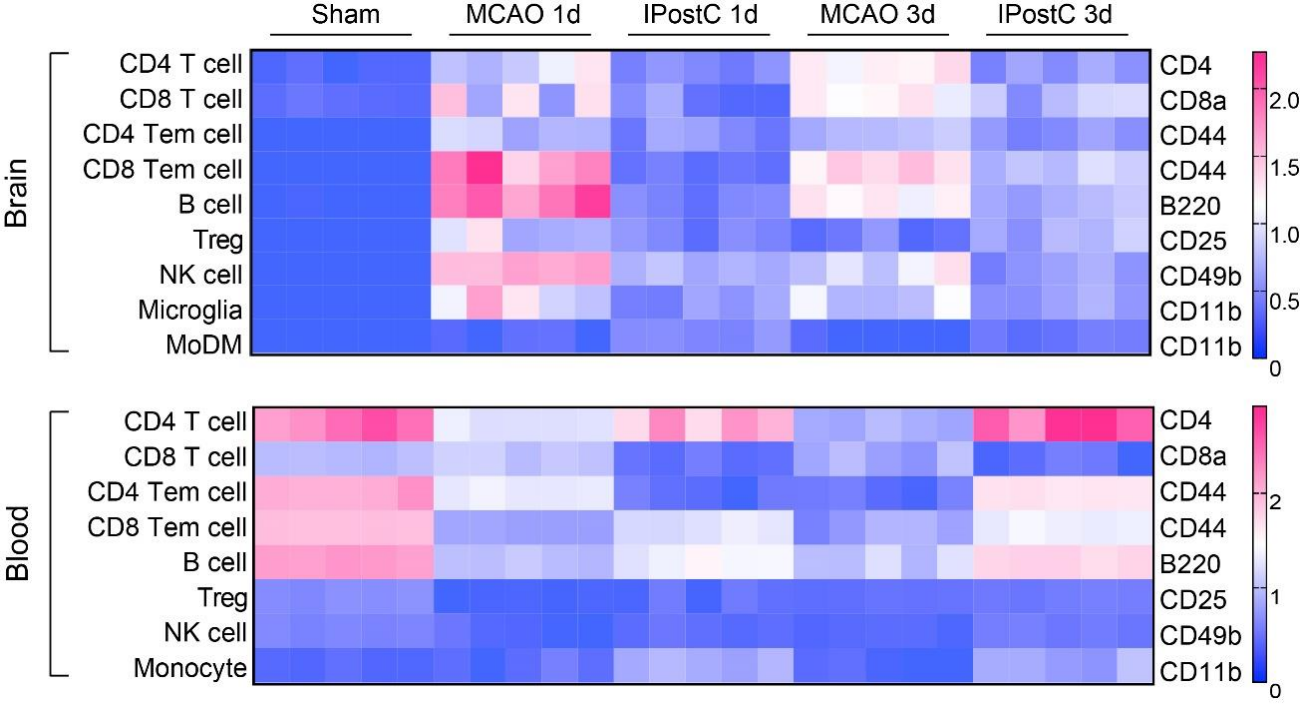
Changes in cell surface markers in different immune cells from samples of sham, MCAO, IPostC at 1, 3 days post-stroke in ischemic brain and peripheral blood. The heatmap depicts the arcsinh ratio of median values. The minimum value in each protein expression was used as a control. The expression levels of cell surface markers include CD11b on Microglia and MoDMs, B220 on B cells, CD4 on CD4^+^T cells, CD8 on CD8^+^T cells, CD44 on CD4 Tem and CD8 Tem cells, CD25 on Tregs, CD11c on cDCs, and CD49b on NK cells. MoDMs, monocyte-derived macrophages; CD4 Tem/CD8 Tem cell, effector memory CD4 and CD8 T cells; Treg cells, regulatory T cells; cDC, conventional dendritic cells; NK cells, natural killer cells. n = 5/group.

## RESULTS

### Application of mass cytometry for phenotypic and functional analysis of immune cells after IPostC

We used an advanced strategy to enable capture the immune cell subsets within the ischemic brain and peripheral blood after IPostC, as shown in Figure 1. In our study, a 35 antibodies CyTOF panel (Supplementary Table 1) was designed, which used to analyze the cellular phenotypes and signaling events after IPostC. Single cell suspensions of ischemic brain and peripheral blood from Sham, MCAO or IPostC group at day 1 and day 3 after stroke (Fig. 1B), and then were stained with the antibody panel. All antibodies used in this study have been validated for commercial purposes and titrated in our experiments. The mass-tag barcoding system was used to make multiplexed samples to reduce sample variations (Fig.1C). The barcoded samples were introduced into the CyTOF, the probes are separated on the basis of their mass to charge ratio, and a mass spectrum that represents the identify and quantity of each isotopic probe vs time resolution was detected (Fig.1D). Since variations in instrument performance can occur across a series of sample runs or even within a single run, the acquired data were calibrated by the MATLAB normalization method (Fig.1E). After debarcoding, the results were analyzed using Cytobank (viSNE and heatmap) and R software (Fig.1F). Several cell phenotype markers were used to identify immune cell subsets in the ischemic brain after IPostC. There is a strategy of analyzing CD45 expression to distinguish resident and infiltrating immune cells, which is higher expression on infiltrating immune cells. In the CD45^low^cells, Microglia (CD45^low^CD11b^+^) was identified based on the expression of CD11b. The remaining CD45^low^ cells were termed ‘undefined cells’ (CD45^low^CD11b^-^). CD45 is also used to identify infiltrating myeloid cells (CD45^high^CD11b^+^) in the ischemic brain. Multiple immune cell subsets were also present in the peripheral blood, including B220^+^ B cells, CD4 T cells, CD8 T cells, CD4 Tem cells, CD8 Tem cells, Tregs, NK cells, cDC and Monocytes.

**Figure 5.**
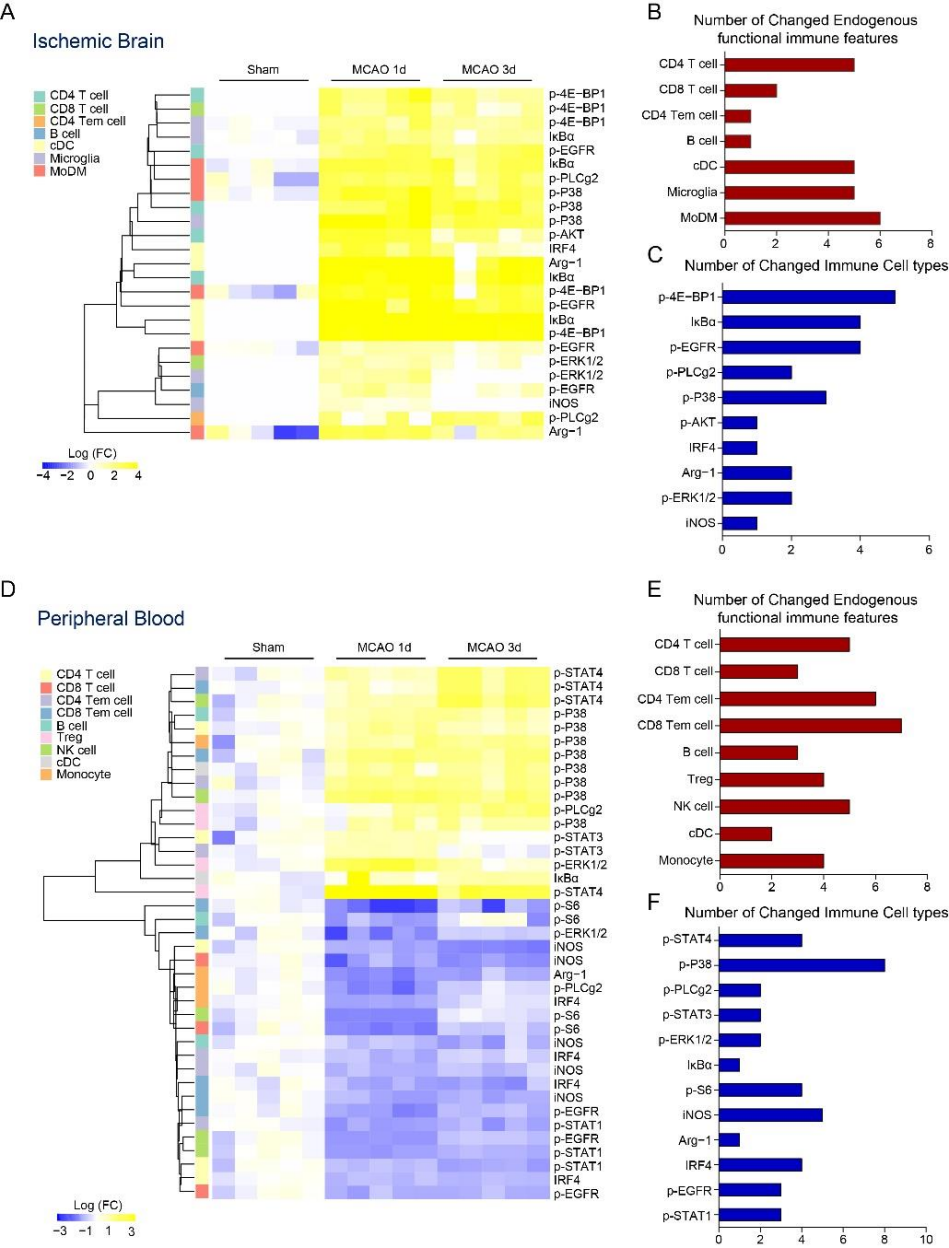
Immune signaling events response to ischemic stroke. (A) The heatmap represents robust changes of various functional proteins in identified immune cell subsets at 1, 3 days after stroke in the ischemic brain. The expression changes were normalized to the mean value of Sham group, with hierarchical clustering by distance using the R package. Functional proteins with significant differences (two-fold, p adjust <0.01) between Sham and MCAO group were selected (B) The bar graph shows the numbers of functional proteins with robust changes in different immune cell subsets in the ischemic brain. (C) The bar graph represents numbers of different immune cell subsets with robust changes of functional proteins in the ischemic brain. (D) The heatmap represents robust changes of various functional proteins in identified immune cell subtypes at 1, 3 days after stroke in the peripheral blood. (E) The bar graph shows numbers of functional proteins with robust changes in different immune cell subsets in the peripheral blood. (F) The bar graph represents numbers of different immune cell subsets with robust changes of functional proteins in the peripheral blood.

**Figure 6.**
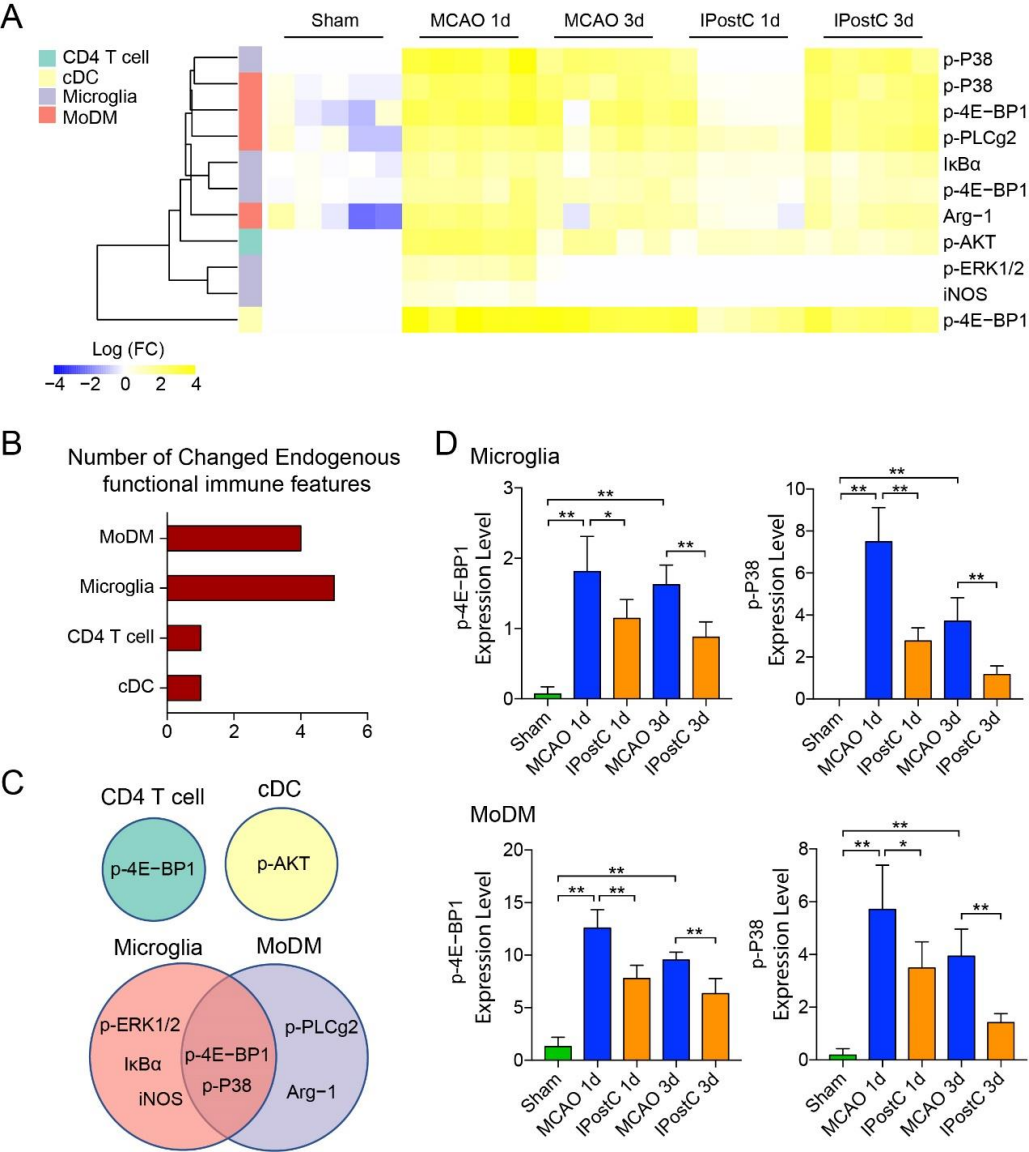
Immune signaling events response to IPostC in the ischemic brain. (A) The heatmap shows significant changes of functional proteins in different immune cell subsets at day 1 and day 3 after stroke or IPostC. The expression changes were normalized to the mean value of Sham group on the heatmap, with hierarchical clustering by distance using R package. Functional proteins with significant differences (two-fold, p adjust <0.01) between MCAO and IPostC group were selected. (B) The bar graph represents the number of functional proteins that are significantly changed in different immune cell subsets in the ischemic brain at 1, 3 days after IPostC. (C) The diagram shows functional proteins with significant changes in different immune cell subsets. (D) Bar graphs show the expression levels of p-4E-BP1 and p-P38 in Microglia and MoDM. *, **, P<0.05, 0.01, respectively, between the two indicated groups. n=5/group.

### IPostC alters immune cells composition in ischemic brain and peripheral blood

First, we performed a viSNE analysis to capture a broad overview of the immune cell subsets at day 1 and day 3 after IPostC. It generates a two-dimensional map where cells with similar multidimensional phenotypes are placed close to each other while maintaining single-cell resolution [22]. To ensure a similar impact of the cells on the viSNE analysis, the number of cells incorporated from the ischemic brain and peripheral blood were matched. Application of viSNE algorithm to the live CD45^+^ events and gating based on distribution of cell markers expression, including CD45, CD11b, CD3e, TCRβ, CD4, CD8a, B220, CD44, CD25, CD49b, CD11c, Ly6G, Ly6C, F4/80. (Fig. 2A, 3A). Indeed, approximately 10 immune cell subsets were defined in ischemic brain and peripheral blood (Fig. 2B, 3B). The heatmap of cell markers (Fig. 2C, 3C) displays how they are expression and intensity difference between the immune cell subsets. However, compared to MCAO group, IPostC significantly decreased the percentage of CD4 T cells, CD4 Tem cells, cDC, and MoDM in the ischemic brain at day 1 (Supplemental Fig.1A) and day 3 after stroke (Fig. 2D). We found that IPostC robustly increased the percentage of CD4 T cells, CD8 Tem cells, B cells, and monocytes compared to MCAO group in the peripheral blood at day 1 (Supplemental Fig.1B) and day 3 after (Fig. 3D). The number of immune cell subsets was also quantified (Supplemental Fig.2, 3). These results demonstrate IPostC inhibited the infiltration of immune cells in the ischemic brain. In addition, IPostC robustly attenuated peripheral lymphopenia and thus improved systemic immunodepression, which is consistent with our previous studies.

### IPostC alters the functional cell surface makers expression

Cell surface markers useful for the identification and characterization of immune cell subsets. Some cell surface markers play a key role in intercellular communication and recognition, which have the task of allowing the transport of molecules across the membrane. Previous study has shown that hypoxic-ischemic encephalopathy induced CD11b expression on circulating Gr-1 positive cells [24].The expression of these cell surface markers in multiple immune cells was also influenced by stroke. To compare the different expression among the various cell subsets at day 1 and day 3 after IPostC (Fig.4), we summarized these results in the two compartments. The heatmap depicts the arcsinh ratio of median values. The minimum value in each protein expression was used as a control. The cell surface markers include CD11b expression on Microglia and MoDMs, B220 expression on B cells, CD4 expression on CD4 T cells, CD8 expression on CD8 T cells, and CD44 expression on CD4 Tem and CD8 Tem cells. CD25 expression on Tregs. CD11c expression on cDCs. CD49b expression on NK cells. The results also showed the expression of CD4, CD8a, CD44, B220, CD49b, CD11b in relevant immune cell populations was all upregulated at day 1 and day 3 after stroke in the ischemic brain, however, IPostC decreased their expressions, which signify their unique roles in specific cell population and need more investigations. Comparable data obtained from peripheral blood also showed that the expression densities of some cell markers in relevant immune populations were influenced by IPostC. These results demonstrate the expression of cell surface marker reflects the degree of inflammation in different tissues, which manipulate the functions of immune cells and further influence brain injury.

### Immune signaling events response to stroke in ischemic brain and peripheral blood

In our study, the intracellular responses were assessed by measuring immune signaling events. The total protein levels of regulatory factors (IκBα) , the phosphorylation of key kinases (including p-PLCg2, p-ERK1/2, p-EGFR, p-P38, p-AKT, p4E-BP1 and p-S6) and transcription factors (p-STAT1, p-STAT3, p-STAT4, IRF4, IRF5, Tbet, Gata3) were involved. Results shown in Fig. 5A are the differences in endogenous signaling in response to stroke, respectively. It seems that all the signaling events increased in the ischemic brain (Fig.5 A-C), including p-4E-BP1, p-EGFR, p-AKT, p-P38, IκBα, Arg-1 were observed striking increased in CD4 T cells. In CD8 T cells, p-4E-BP1 and p-ERK1/2 were increased. p-4E-BP1, p-EGFR, IκBα, IRF4 were increased in cDCs. In microglia, p-4E-BP1, p-ERK1/2, p-P38, IκBα, iNOS were observed increased. p-4E-BP1, p-EGFR, p-PLCg2, p-P38, IκBα, Arg-1 were observed increased in MoDMs. The expression of p-PLCg2 in CD4 Tem cells and pEGFR in B cells were significantly increased (Supplemental Table. 2-3).

In the peripheral blood, the top 12 immune signaling events split into 4 events that were elevated and 6 events that were decreased in all cell subtypes after stroke. 2 features were elevated or decreased in some cell subtypes (Fig.5D-F). The evoked p-P38 signal was visibly increased in CD4 Tem cells, CD8 Tem cells, Tregs and NK cells compared with those in MCAO group. The expression level of p-STAT4 signal increased in CD4 T cells, CD4 Tem cells, CD8 Tem cells, B cells, Tregs, NK cells, cDCs and monocytes was dramatically increased after stroke. The evoked p-STAT3 was increased in CD4 T and CD4 Tem cells. The expression of p-ERK1/2 and IκBα increased in Tregs and cDCs respectively. The analysis revealed that endogenous p-pS6 signals were decreased in CD8 T cells, CD8 Tem cells, B cells, NK cells subsets. A decrease expression of iNOS was observed in multiple adaptive immune cell subsets, including CD4 T cells, CD8 T cells, CD4 Tem cells, CD8 Tem cells and B cells subsets. IRF4 was significantly decreased in CD4 T cells, CD4 Tem cells, CD8 Tem cells and monocytes. p-EGFR was decreased in CD8 T cells, CD8 Tem and NK cells. p-STAT1 was decreased in CD4 T cells, CD4 Tem and NK cells. p-ERK1/2 was decreased in CD8 Tem cells. p-PLCg2 and Arg-1 were decreased in monocytes (Supplemental Table. 2-3).

**Figure 7.**
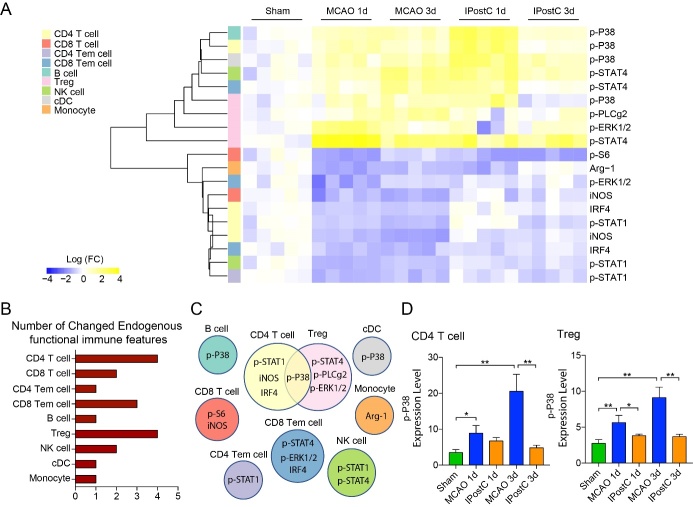
Immune signaling events response to IPostC in the peripheral blood. (A) The heatmap compares significant changes of functional proteins in different immune cell subsets at day 1 and day 3 after stroke or IPostC. The expression changes were normalized to the mean value of the Sham group and were hierarchically clustered by distance using R package. Functional proteins with significant differences (two-folds, p adjust <0.01) between the MCAO and IPostC groups were selected. (B) The bar graph shows the number of significantly changed functional proteins in different immune cell subsets in the peripheral blood at 1, 3 days after IPostC. (C) The diagram indicates the names of significantly changed functional proteins in different immune cell subsets. (D) Bar graphs represent the expression levels of P38 in CD4 T cells and Treg cells, respectively. *, **, P<0.05, 0.01, respectively, between the two indicated groups. n=5/group.

### Downregulating of 4E-BP1 and P38 in Microglia and MoDM response to IPostC

Differences in signaling responses to IPostC were identified and compared to the sham group according to signal intensities. In response to IPostC, 8 immune signaling events were decreased compared to MCAO group (Fig. 6A). Compliance with signaling activities, 8 events related to signaling responses in immune cell subsets were elevated after stroke, that is, p-4E-BP1, p-P38, p-ERK1/2, IκBα and iNOS in Microglia. p-4E-BP1, p-P38, p-PLCg2 and Arg-1 in MoDMs. p-4E-BP1 in CD4 T cells and p-AKT in cDCs (Fig.6B). Particularly, a decrease in p-P38 and p-4E-BP1 signals were observed across microglia and MoDM (Fig.6C-D). This suggests that Microglia and MoDM are key cell types related to mechanisms regulate the activity during IPostC. IPostC induced protection is involved in downregulating 4E-BP1 and P38 of Microglia and MoDM after stroke.

### Downregulating P38 in CD4 T cell and Treg response to IPostC

In the peripheral blood, 9 immune signaling events were decreased response to IPostC (Fig.7A)., including p-P38, p-ERK1/2, p-PLCg2, p-S6, p-STAT1, p-STAT4, IRF4, iNOS and Arg-1. p-P38 was observed striking decreased in CD4 T cells, B cells, Tregs and cDCs. In CD8 Tem cells, p-ERK1/2 was increased response to IPostC. In contrast, p-ERK1/2 was decreased. P-STAT4 in CD8 Tem cells, Tregs and NK cells were observed decreased. P-STAT1 in CD4 T cells, CD4 Tem cells and NK cells were increased. IRF4 was increased in CD4 T cells and CD4 Tem cells. iNOS were observed increased in CD4 and CD8 T cells. P-S6, Arg-1 were observed increased in CD8 T cells and MoDMs respectively. The expression of p-PLCg2 in Tregs was significantly decreased (Fig.7B). This suggests that CD4 T cell and Treg are crucial cell types in the peripheral blood during IPostC. IPostC induced protection is involved in downregulating P38 of CD4 T cell and Treg after stroke (Fig.7C-D). Yet, when taken together, the signaling events P38, a critical pattern in ischemic brain and peripheral blood after IPostC, are broadly blunted across immune cells.

**Figure 8.**
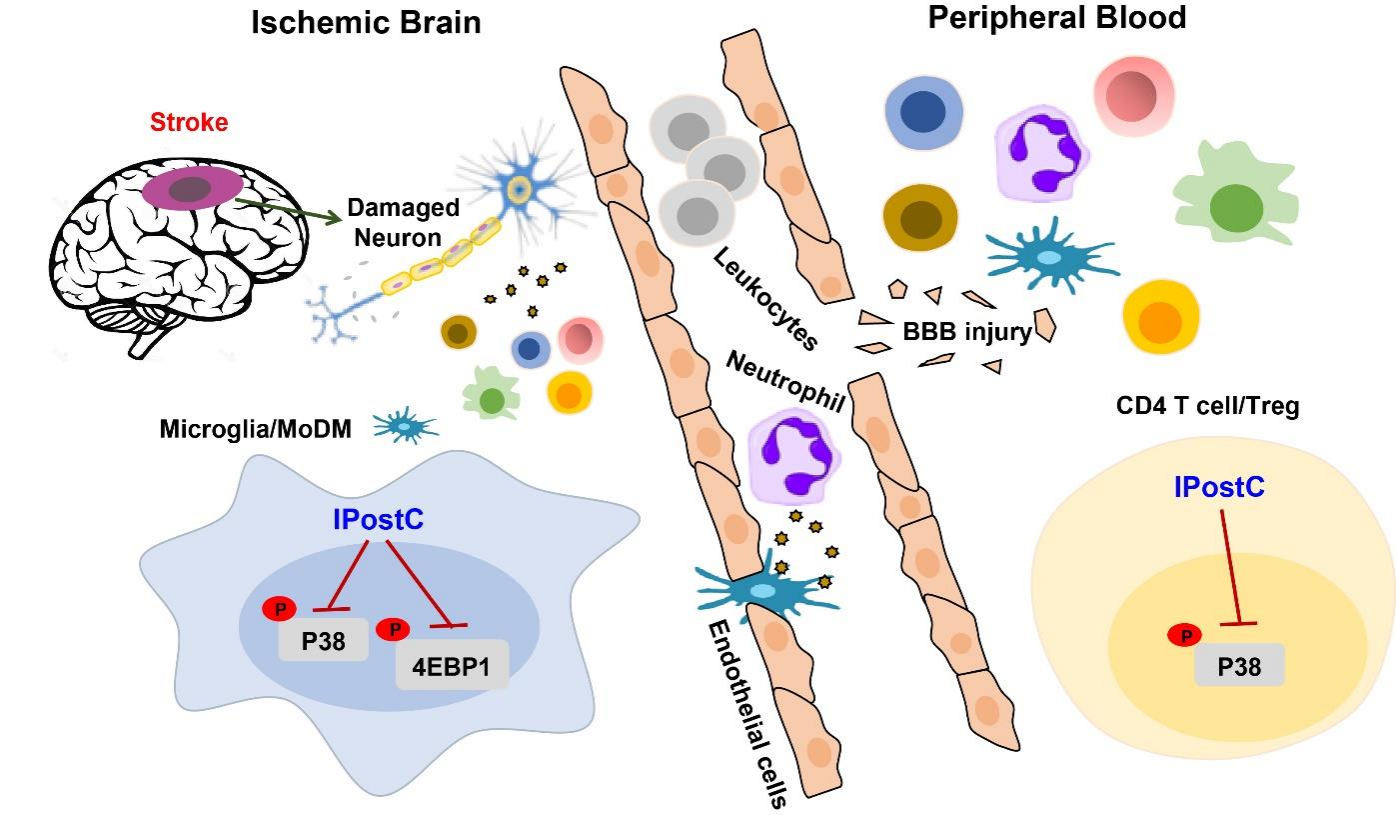
Our results showed that downregulation of 4E-BP1 and p38 of Microglia and MoDM in the ischemic brain was involved in IPostC-induced protection. In the peripheral blood, downregulation of P38 of CD4 T cell and Treg has also participated in IPostC-induced protection.

## DISCUSSION

After ischemic stroke, brain injury triggers a series of robust inflammatory response, leading to the activation and infiltration of inflammatory cells into the ischemic brain. [25-28]. Most literature indicates that inflammation promotes the expansion of stroke lesions and worsens neurodeficits during stroke acute phase [29, 30]. Further, inhibit the immune response in the brain can limit the extent of stroke injury [26, 27, 31-33]. Contrary, the injured brain can transition the functional status of the peripheral immune system from competence to suppression after acute stroke, as manifested by lymphopenia, decreased the levels of inflammatory cytokines, monocyte and lymphocyte dysfunction [29, 34, 35].

The sudden break of blood supply in the vascular region results in neural cells' death, engendering an ischemic core, surrounded by a hypoperfused region termed the penumbra [36]. Focal cerebral ischemia leads to microglia are activated in the cerebral cortex of the ischemic hemisphere. Microglia are the first cell populations in the CNS to react to these danger signals within minutes of ischemia onset [25]. Astrocytes actively participate in the immune response after injury by recruiting peripheral immune cells and interacting with microglia via secreting cytokines and chemokines [37, 38]. Following ischemia, dynamic blood-brain barrier (BBB) permeability changes result in endothelial swelling [39], and invasion of peripheral myeloid cells and lymphocytes drive inflammation progression. This leads to the production of adhesion molecules and proinflammatory cytokines, which in turn aggravates the damage of brain parenchyma and vasculature [40].

In a murine MCAO model, myeloid cells and lymphocytes were identified in the contralateral hemisphere throughout acute and chronic phases, which is continuously at least 2 weeks after stroke [41, 42]. Neutrophils are attracted to the activated endothelium and reach perivascular space, which is detected in the microvessels within the first hour after ischemic stroke. Alternatively, other inflammatory cells such as macrophages, lymphocytes, and dendritic cells are infiltrated in the ischemic hemisphere. Attracted by specific chemokines, immature proinflammatory monocytes infiltrate the ischemic tissue where they mature into macrophages and are involved in tissue repair after acquiring alternative polarization signs. Current experimental evidence suggests that lymphocytes, in particular, accumulation of T cells within the thalamus, seem to play innate functions and interact with players in thrombosis and hemostasis in the acute phase of stroke [42]. Experimental studies found that induced persistence of NK cells in the ischemic tissue achieved by interfering with nicotinic acetylcholine receptor did not modify lesion size but increased systemic interferon γ, protected from bacterial infection, and enhanced post-stroke survival [35].

For many years, flow cytometry is the main technique of immune cell phenotyping in the ischemic brain, which is allowing simultaneous detection of several antigens. But the significant challenges of flow cytometry are tissue autofluorescence and spectral overlap. However, CyTOF offers a significant advance in stroke research, which allows high-dimensional multi-parameter analysis [43]. Here, we use this technique can detect the surface-marker expression of in various immune cell subsets at the single-cell level after IPostC.

Our results demonostrate IPostC inhibited the infiltration of immune cells in the ischemic brain. In addition, IPostC robustly attenuated peripheral lymphopenia, which is consistent with our previous studies. Also, we found that the expression of cell surface marker reflects the degree of inflammation, which manipulate the functions of immune cells and further influence brain injury. In the current study, CyTOF was used to study neuroinflammation after IPostC. With this high dimensional technique for single-cell analyses, we identified 10 phenotypes of leukocytes recruited in the ischemic brain. We find that IPostC strongly inhibits the phosphorylation of 4EBP1 and P38 in Microglia and MoDM in the ischemic brain.

After acute cerebral ischemia, activated microglia response to the influx of immune cells and cellular damage, which release cytotoxic and cytoprotective substances. In ischemic brain tissue, infiltrated monocyte-derived macrophages are key modulators of the immune system. Resident microglia and MoDM are involved in the production of inflammatory cytokines, such as transforming necrosis factor-α (TNF-α), interleukin-1β (IL-1β), and transforming growth factor-β (TGF-β). Several studies have reported that microglia and MoDM induce neuronal injury in the acute phase of ischemic stroke through a TLR-4-dependent manner and trigger the proinflammatory mediator. Pharmacological inhibition of microglia showed protective effects in cerebral ischemia by inhibiting a microglia-derived inflammatory mediator. Most recently, we demonstrated that the mTOR cell signaling pathway contributes to brain damage after IPostC. We measured some of the phosphorylated proteins associated with the mTOR pathway in the ischemic hemisphere, including S6K1, S6, and 4EBP1. IPostC attenuated reductions in their levels after stroke. Furthermore, mTOR inhibition, both by the mTOR inhibitor rapamycin and mTOR shRNA, aggravated ischemic outcomes and eliminated the protective effect of IPostC [44].

During acute diseases, inflammatory mediators released, which activate multiple intracellular signaling cascades, including the MAPK signal transduction pathway. It is an important intracellular signal transduction system and plays a crucial role in the recruitment of leukocytes to inflammation sites. Stimulated leukocytes by pro-inflammatory cytokines result in the activation of MAPK isoform p38. However, the functional consequences of p38 MAPK activation, including adhesion, migration, and effector functions are just beginning to be elucidated [45-47]. The leading members of MAPK family are extracellular signal-regulated protein kinase (ERK1/2), c-Jun N-terminal protein kinase (JNK), and p38 MAPK. Many studies showed that p38 MAPK plays a role in the protective effects of ischemic preconditioning. However, some reports indicated that the activation of p38 MAPK might closely be associated with the regulation of inflammation and oxidative stress. Microglial phagocytosis in living slice cultures was inhibited by the p38MAPK inhibitor SB203580 [48]. The survival and phagocytosis of activated microglia in vitro were promoted by MAPKs, including p38 [49].

From a clinical perspective, IPostC appears to be attractive as it can be potentially translated, in particular for patients subjected to endovascular therapies. A large amount of data supports its beneficial effects in neurons. Our study provides proof-of-concept that IPostC may also modify immune cell subsets’ crosstalk (Fig. 8).

A limitation of our study is that most of neutrophils were excluded in the ischemic brain with the current cell isolation protocol. CyTOF does not provide the information of position, which may be essential for a particular cell phenotype. However, imaging mass cytometry [50] could be valuable to address this issue. The panel of antibodies in our study has been validated, which could be useful for imaging CyTOF in the future. The advantage of CyTOF is to address multiple cell phenotyping and intracellular signaling events after IPostC. Increased understanding of the function of cellular subsets and intracellular signaling events will enable us to develop more selectively targeted therapies.For instance, only young male adult animals were used in our study; nevertheless, animal age and sex are important factors that significantly affect stroke outcomes and neuroinflammation [51, 52]. Therefore, future experiments should be conducted by including female and aged animal models.

## Supplementary Materials

The Supplemenantry data can be found online at: www.aginganddisease.org/EN/10.14336/AD.2020.1115.


