# Supplementary material for: Systematic Study of Immune Cell Diversity in ischemic postconditioning Using High-Dimensional Single-Cell Analysis with Mass Cytometry
Source: Aging Dis. 2021 Jun 1;12(3):812–25. doi: 10.14336/AD.2020.1115 (PMC8139206; doi:10.14336/AD.2020.1115)
Supplement: Supplementary file 1 [file AD-12-3-812-s.pdf]

SUPPLEMENTARY DATA

Systematic Study of Immune Cell Diversity in ischemic postconditioning Using High-Dimensional Single-Cell Analysis with Mass Cytometry

Yang Yao[1*], Yaning Li[1], Weihua Ni[1], Zhijun Li[2], Liangshu Feng[1], Yan Wang[1], Jihong Meng[1], Heng Zhao[1*]

[1]Department of Neurosurgery, Stanford University School of Medicine, Stanford, CA 94305, USA
[2]Division of Plastic and Reconstructive Surgery, Department of Surgery, Stanford University School of Medicine, Stanford, CA 94305, USA

© 2020. Yao Y et al. Published online at http://www.aginganddisease.org/EN/10.14336/AD.2020.1115

SUPPLEMENTARY DATA

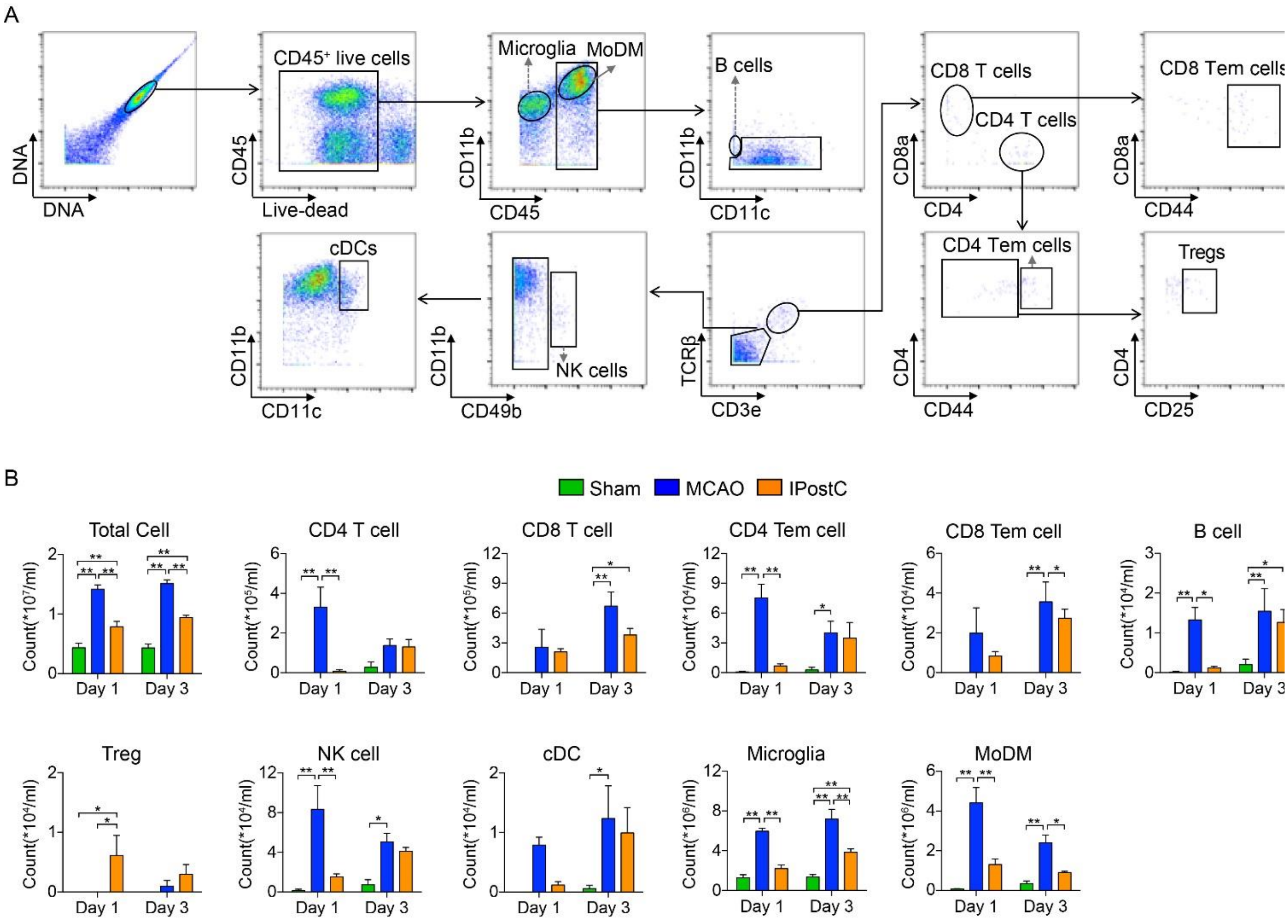

Supplementary Figure 1. Bar graphs show the percentage of cell populations identified in the viSNE clustering at day 1 after stroke. Data are presented as Mean ± SD, and dots on the bars represent individual samples. MoDMs, monocyte-derived macrophages; CD4 Tem/ CD8 Tem cell, effector memory CD4 and CD8 T cells. Treg, regulatory T cells; cDC, conventional dendritic cells; NK cells, natural killer cells. n=5/group. *, **, P<0.05, 0.01, respectively, between the two indicated groups.

© 2020. Yao Y et al. Published online at http://www.aginganddisease.org/EN/10.14336/AD.2020.1115

SUPPLEMENTARY DATA

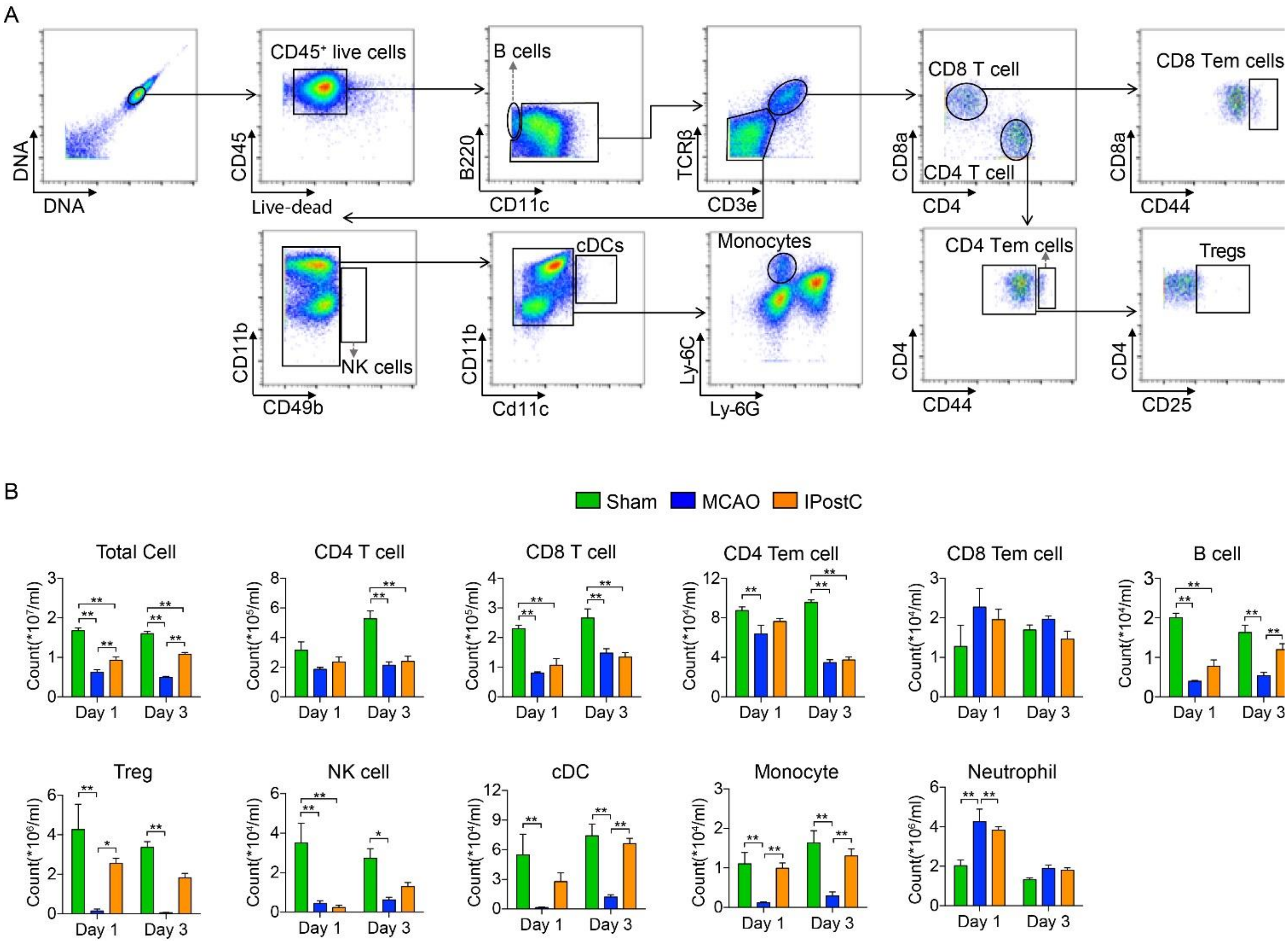

Supplementary Figure 2. Analysis of inflammatory cells in the ischemic brain. (A) Manual gating strategies for diverse immune cell types in samples of the ipsilateral ischemic hemisphere 3 days after stroke. (B) Quantitative data of the total number of immune cells and their subtypes were identified from manual gating strategy.

© 2020. Yao Y et al. Published online at http://www.aginganddisease.org/EN/10.14336/AD.2020.1115

SUPPLEMENTARY DATA

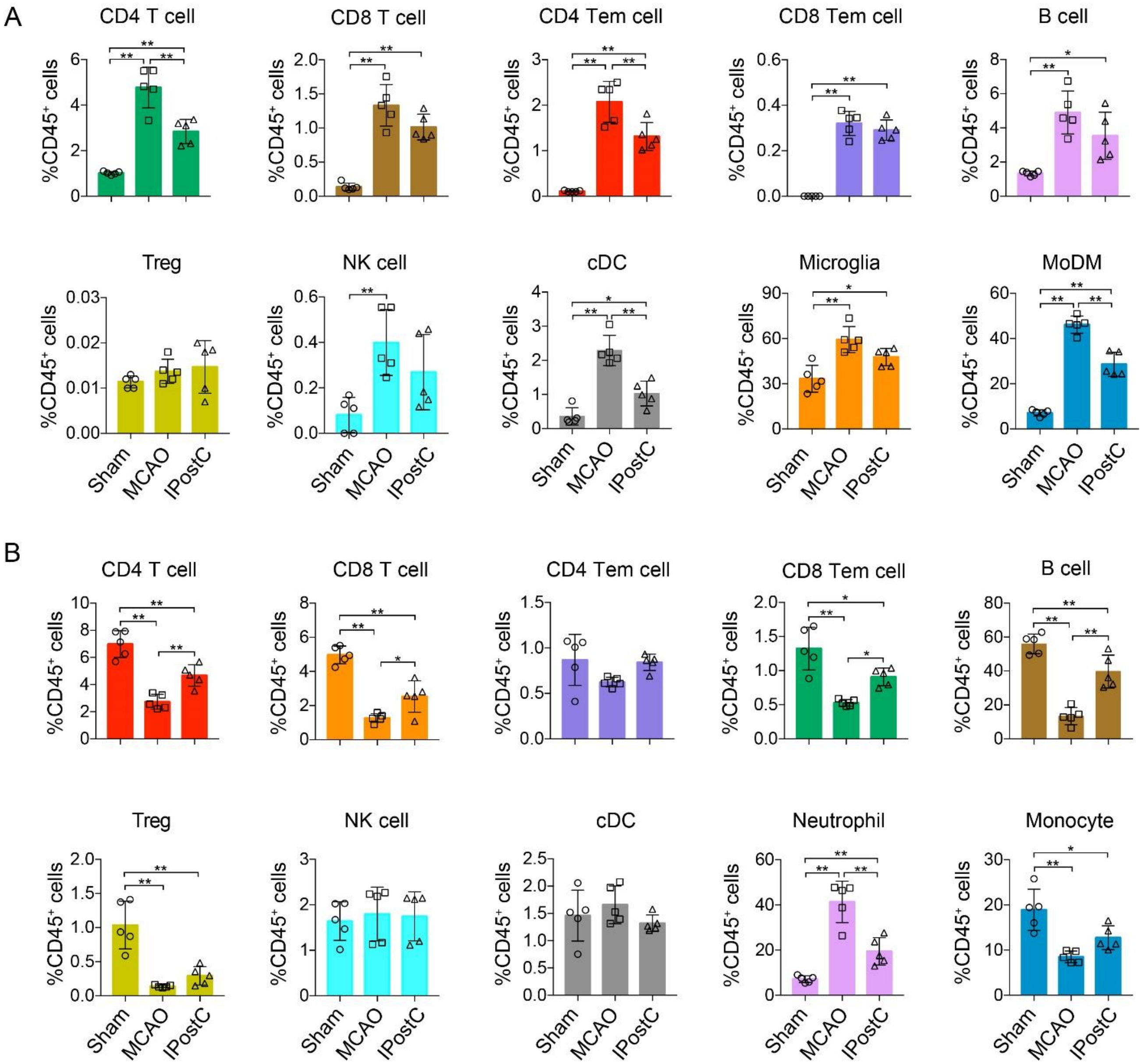

Supplementary Figure 3. Analysis of peripheral blood leukocytes after stroke. (A) Manual gating strategies for immune cell subsets in peripheral blood samples collected 3 days after stroke. (B) Quantitative data of the total number of immune cells and their subtypes were identified from manual gating strategy.

© 2020. Yao Y et al. Published online at http://www.aginganddisease.org/EN/10.14336/AD.2020.1115

SUPPLEMENTARY DATA

Supplementary Table 1. The panel of Cytof antibody.

CyTOF antibody panel. The full metal-conjugated antibody panel. Used for CyTOF experiments. Amount of each antibody is referred to one sample with $3*10^6$ cells, at a 100µl final staining volume.

Metal isotope	Antibody	Host	Amount(µl)	Clone	Source	Cat.#
**141Pr**	Ly6G	Mouse	0.5	1A8	Fluidigm	3141005B
**142Nd**	CD11c	Mouse	0.5	N418	Fluidigm	3142003B
**143Nd**	CD357	Mouse	0.5	DTA1	Fluidigm	3143019B
**145Nd**	CD45RB	Mouse	0.5	C363.16A	Fluidigm	3145012B
**147Sm**	CD45	Mouse	0.5	30-F11	Fluidigm	3147003B
**148Nd**	CD11b	Mouse	0.5	M1/70	DVS Science	3148003B
**150Nd**	CD44	Human/Mouse	0.5	IM7	DVS Science	3150018B
**151Eu**	CD25	Mouse	0.5	3C7	Fluidigm	3151007B
**154Sm**	CD48	Mouse	0.5	HM48-1	DVS Science	3154004B
**159Tb**	F4/80	Mouse	0.5	BM8	DVS Science	3159009B
**162Dy**	Ly6C	Mouse	0.5	HK1.4	Fluidigm	3162014B
**163Dy**	CD54	Mouse	0.5	YN1/1.7.4	Fluidigm	3163020B
**165Ho**	CD3e	Mouse	0.5	145-2C11	DVS Science	3165020B
**168Er**	CD8a	Mouse	0.5	53-6.7	Fluidigm	3168003B
**169Tm**	TCRβ	Mouse	0.5	H57-597	Fluidigm	3169002B
**170Er**	CD49b	Mouse	0.5	HMa2	DVS Science	3170008B
**172Yb**	CD4	Mouse	0.5	RM4-5	Fluidigm	3172003B
**176Yb**	CD45R/B220	Human/Mouse	0.5	RA3-682	DVS Science	3176002B
**144Nd**	pPLCg2	Mouse	0.5	K86-689.37	DVS Science	3144015A
**171Yb**	pERK1/2	Mouse	0.5	D13,14,4E	Fluidigm	3171010A
**146Nd**	pEGFR	Mouse	0.5	D7A5	Fluidigm	3146007A
**156Gd**	p-p38	Mouse	0.5	T180/Y182	Fluidigm	3156002A
**153Eu**	pStat1	Mouse	0.5	4a	Fluidigm	3153005A
**174Yb**	pStat4	Mouse	0.5	38	DVS Science	3174005A
**158Gd**	pStat3	Mouse	0.5	4	Fluidigm	3158005A
**152Sm**	pAKT	Mouse	0.5	D9E	Fluidigm	3152005A
**149Sm**	p4E-BP1	Mouse	0.5	23684	Fluidigm	3149005A
**175Lu**	pS6	Mouse	0.5	S235/S236	DVS Science	3175009A
**155Gd**	IRF-4	Human/Mouse	0.5	3E4	Fluidigm	3155014B
**173Yb**	IRF-5	Mouse	0.5	10T1	Fluidigm	Not applicable
**161Dy**	iNOS	Mouse	0.5	CXNFT	Fluidigm	3161011B
**166Er**	Arg-1	Mouse	0.5	Polyclonal	Fluidigm	3166023B
**160Gd**	Tbet	Human/Mouse	0.5	4B10	Fluidigm	3160010B
**167Er**	Gata3	Human/Mouse	0.5	TWAJ	Fluidigm	3167007A
**164Dy**	IkBa	Mouse	0.5	L35A5	Fluidigm	3164004A

SUPPLEMENTARY DATA

Supplementary Table 2. Identified immune cell types and their identifying markers.

**Ischemic brain**		**Peripheral blood**	
**Cell Types**	**Defined Markers**	**Cell Types**	**Defined Markers**
Microglia	$CD45^{low}CD11b^{+}$	Monocytes	$CD45^{+}Ly6C^{+}Ly6G^{-}$
Monocyte derived macrophages (MoDM)	$CD45^{high}CD11b^{+}$	B cells	$CD45^{+}B220^{+}$
B cells	$CD45^{high}B220^{+}$	CD4 T cells	$CD45^{+}TCR\beta^{+}CD3^{+}CD4^{+}$
CD4 T cells	$CD45^{high}CD3^{+}CD4^{+}$	CD8 T cells	$CD45^{+}TCR\beta^{+}CD3^{+}CD8^{+}$
CD8 T cells	$CD45^{high}CD3^{+}CD8^{+}$	CD4 Tem cells	$CD45^{+}TCR\beta^{+}CD3^{+}CD4^{+}CD44^{+}$
CD4 Tem cells	$CD45^{high}CD3^{+}CD4^{+}CD44^{+}$	CD8 Tem cells	$CD45^{+}TCR\beta^{+}CD3^{+}CD8^{+}CD44^{+}$
CD8 Tem cells	$CD45^{high}CD3^{+}CD8^{+}CD44^{+}$	Tregs	$CD45^{+}TCR\beta^{+}CD3^{+}CD4^{+}CD25^{+}$
Tregs	$CD45^{high}CD3^{+}CD4^{+}CD25^{+}$	NK cells	$CD45^{+}CD3^{-}CD49b^{+}$
NK cells	$CD45^{high}CD3^{-}CD49b^{+}$	cDC	$CD11b^{+}CD11c^{+}$
cDC	$CD11b^{+}CD11c^{+}$		

© 2020. Yao Y et al. Published online at http://www.aginganddisease.org/EN/10.14336/AD.2020.1115

Supplementary Table 3. Changed endogenous functional immune features in different cell types.

Ischemic Brain				
**CD4 T cell**	**CD8 T cell**	**CD4 Tem cell**	**B cell**	**cDC**
p-P38<br>p-4E-BP1<br>p-EGFR<br>p-AKT<br>IκBα	p-P38<br>p-ERK1/2	p-PLCg2	p-EGFR	p-4E-BP1<br>p-EGFR<br>IκBα<br>Arg-1<br>IRF4
**Microglia**	**MoDM**			
p-4E-BP1<br>p-P38<br>p-ERK1/2<br>IκBα<br>iNOS	p-4E-BP1<br>p-P38<br>p-EGFR<br>p-PLCg2<br>Arg-1<br>IκBα			
**Peripheral Blood**				
**CD4 T cell**	**CD8 T cell**	**CD4 Tem cell**	**CD8 Tem cell**	**B cell**
p-P38<br>p-STAT1<br>p-STAT3<br>IRF4<br>iNOS	p-EGFR<br>p-S6<br>iNOS	p-P38<br>p-STAT1<br>p-STAT3<br>p-STAT4<br>iNOS<br>IRF4	p-P38<br>p-STAT3<br>p-STAT4<br>p-ERK1/2<br>p-EGFR<br>p-S6<br>IRF4	p-P38<br>p-S6<br>iNOS
**Treg**	**NK cell**	**cDC**	**Monocyte**	
p-P38<br>p-ERK1/2<br>p-PLCg2<br>p-STAT4	p-P38<br>p-EGFR<br>p-STAT1<br>p-STAT4<br>p-S6	p-P38<br>IκBα	p-P38<br>p-PLCg2<br>Arg-1<br>IRF4	

© 2020. Yao Y et al. Published online at http://www.aginganddisease.org/EN/10.14336/AD.2020.1115

SUPPLEMENTARY DATA

Supplementary Table 4. Changed cell types in different endogenous functional immune features.

**Ischemic Brain**	
**p-4E-BP1**	CD4 T cell, CD8 T cell, cDC, Microglia, Monocyte
**p-P38**	CD4 T cell, Microglia, Monocyte
**p-EGFR**	CD4 T cell, B cell, cDC, Monocyte
**p-PLCg2**	CD4 Tem cell, Monocyte
**p-ERK1/2**	CD8 T cell, Microglia
**p-AKT**	CD4 T cell
**IκBα**	CD4 T cell, cDC, Microglia, Monocyte
**iNOS**	Microglia
**Arg-1**	cDC, Monocyte
**IRF4**	cDC
**Peripheral Blood**	
**p-P38**	CD4 T cell, CD4 Tem cell, CD8 Tem cell, B cell, Treg, NK cell, cDC, Monocyte
**p-STAT3**	CD4 T cell, CD4 Tem cell
**p-STAT4**	CD4 Tem cell, CD8 Tem cell, Treg, NK cell
**p-PLCg2**	Treg
**p-ERK1/2**	Treg
**IκBα**	cDC
**p-S6**	CD8 T cell, CD8 Tem cell, B cell, NK cell
**p-ERK1/2**	CD8 Tem cell
**p-PLCg2**	Monocyte
**p-EGFR**	CD8 T cell, CD8 Tem cell, NK cell
**p-STAT1**	CD4 T cell, CD4 Tem cell, NK cell
**iNOS**	CD4 T cell, CD8 T cell, CD4 Tem cell, CD8 Tem cell, B cell
**Arg-1**	Monocyte
**IRF4**	CD4 T cell, CD4 Tem cell, CD8 Tem cell, Monocyte

© 2020. Yao Y et al. Published online at http://www.aginganddisease.org/EN/10.14336/AD.2020.1115
